# The benefits and struggles of FAIR data: the case of reusing plant phenotyping data

**DOI:** 10.1038/s41597-023-02364-z

**Published:** 2023-07-13

**Authors:** Evangelia A. Papoutsoglou, Ioannis N. Athanasiadis, Richard G. F. Visser, Richard Finkers

**Affiliations:** 1 Plant Breeding, Wageningen University and Research, Wageningen, The Netherlands; 2 Wageningen Data Competence Center and Geo-Information Science & Remote Sensing Lab, Wageningen University and Research, Wageningen, The Netherlands; 3 Present Address: Taxonic B.V., De Meern, The Netherlands; 4 Present Address: GenNovation B.V., Wageningen, The Netherlands

**Keywords:** Research data, Plant breeding

## Abstract

Plant phenotyping experiments are conducted under a variety of experimental parameters and settings for diverse purposes. The data they produce is heterogeneous, complicated, often poorly documented and, as a result, difficult to reuse. Meeting societal needs (nutrition, crop adaptation and stability) requires more efficient methods toward data integration and reuse. In this work, we examine what “making data FAIR” entails, and investigate the benefits and the struggles not only of reusing FAIR data, but also making data FAIR using genotype by environment and QTL by environment interactions for developmental traits in potato as a case study. We assume the role of a scientist discovering a phenotypic dataset on a FAIR data point, verifying the existence of related datasets with environmental data, acquiring both and integrating them. We report and discuss the challenges and the potential for reusability and reproducibility of FAIRifying existing datasets, using metadata standards such as MIAPPE, that were encountered in this process.

## Introduction

Plant phenotyping is an important process underlying breeding, producing plant varieties with improved attributes to meet the needs of a growing population^1^. Phenotyping produces data that is as intricate as the process itself, reflecting the heterogeneity of experimental goals, designs and settings and agronomic management practices. Further complexity is observed because of the many types of data it can involve, assembled by humans and/or machines.

The complexity of phenotypic data is a hindrance to reuse. Like most domains of science, though perhaps to an even greater extend because of the many species involved as well as new high-throughput technologies, plant phenotyping has undergone an explosion in the size and number of datasets. However useful each of them may be to the original producer, their potential to deepen our understanding of plant biology remains unmaterialized because meta-analyses across independently generated datasets are difficult, both epistemologically (ambiguities, missing documentation) and logistically (undiscoverable data, different data types)^2,3^.

To improve the global data landscape, managing the challenges of heterogeneity and attempting to bridge distributed resources, the FAIR (Findable, Accessible, Interoperable, Reusable) data principles have been proposed^4^. Some of their requirements are common across scientific domains, whereas others target a community-wide consensus. The plant phenotyping community has made progress with the MIAPPE metadata standard^5^, which aids three aspects of FAIR: findability (experiments are annotated with relevant, searchable attributes); interoperability (common metadata vocabulary); and reusability (minimum information necessary for interpretation and reuse). Accessibility is tackled through concrete implementations such as the Breeding API^6^.

Without elements of FAIR and supporting standards like MIAPPE, data reuse can be challenging. Conducting a meta-analysis involves reusing primary data collected in previous studies, which is a laborious process: data need to be aggregated from original sources, aligned syntactically and semantically, and potentially linked with other secondary/cofounder data. This often looks like “data archaeology” where original fragments of information need to be carefully stitched together to reconstruct the full picture. FAIR data can be a remedy to this situation, however it is neither a straightforward process, nor an easy one.

To investigate the benefits and the struggles of FAIR data, we re-implemented a meta-analysis using FAIR data infrastructures: How primary data can be annotated with metadata, discovered, aggregated, combined with secondary data, and ultimately explored together. We develop a proof of concept (PoC) implementation to support the steps of discovery, integration and reuse, that relies on FAIR principles, community standards, as MIAPPE, and offer an interactive front-end for query and visualization with Jupyter notebooks. In this process, we report our experience in terms of both technical performance and user experience. We have two goals: one is to evaluate how technically such an exercise can be conducted when FAIR principles and plant phenotyping data standards are used. Second, what are the benefits in terms of time and effort. Both goals are relevant to the plant phenotyping and, in general, the FAIR data communities to assess the efficacy of current solutions and drive future developments.

Without loss of generality, we worked with an example of successful data reuse, the work in Hurtado-Lopez’s doctoral thesis that investigated genotype by environment and QTL by environment interactions for developmental traits in potato^7^. Her meta-analyses relied on five experiments with potato conducted in four different locations across different latitudes, as the environmental variation affects developmental processes. Partially overlapping subsets of the CxE population (a diploid backcross mapping population extensively studied at Wageningen University & Research)^8^ were used for all experiments (including^7,9,10^). Traits of morphological (e.g. tuber size), developmental (e.g. flowering), and agronomic (e.g. yield) nature were evaluated. Additionally, this work integrated different data types, laying a basis for in-depth meta-analyses reusing primary data from a variety of domains (including genetic, phenotypic, molecular and environmental). Among the insights in Hurtado-Lopez’s work, one concerns the effects of temperature and photoperiod on agronomic traits.

It is important to note that the experiments were conducted by different, uncoordinated parties in different time periods (over 11 years), though there was a direct line of communication with them to establish sufficient understanding for data reuse. Sometimes, this can be the critical difference warranting that datasets are indeed compatible, as omitting experimental design or other details in published materials and protocols may be prohibitive for data reuse. Still, a key conclusion made clear in Hurtado-Lopez’s doctoral thesis is that more needs to be done toward improving data practices. In particular, she identifies three elements that should be part of proper documentation for multi-environment studies, if reuse is to be successful: content, origin/source and structure. These elements are part of the MIAPPE standard^5^. It is also key to notice that, although Hurtado-Lopez’s work is an example of successful reuse, it reflects work that was logistically complicated for entirely unscientific reasons.

Hurtado-Lopez completed her work in 2012, before the establishment of the FAIR data principles, though standardization had already taken root in some scientific domains (e.g. MIAME for microarray data). Therefore, the data she received was often disorganized and sometimes lacked important details, demanding time-consuming communications to resolve. In some cases, data could not be used as there was insufficient information. The harmonization of the variety of formats and data file structures was another point to be tackled. Despite that, Hurtado-Lopez was able to reuse a significant part of the different datasets and arrive at novel findings.

More organized documentation would have resolved ambiguities and reduced time spent locating information in unstructured chains of literature. The ability to tackle data integration and manipulation uniformly for all experiments would have been a practical, time-saving asset. These benefits would have affected the outcome of that work, if more data had been usable. Relieving hindrances to data reuse would have saved resources and enabled the researcher to focus on more biological, and fewer data handling challenges.

We envision a scenario where different data handling stages (acquisition, integration, analysis/reuse) can be streamlined and become easily reproducible. In our proof of concept, we FAIRify datasets and explore the process of discovery and integration. We generate descriptive visualizations with the combined data, which can be invaluable for exploratory analyses. Finally, we investigate the challenges and benefits of the approach.

## Results

In our proof of concept we demonstrate how FAIR can be used for a number of tasks necessary for repeating the meta-analysis. Specifically, this involves: (a) discover relevant phenotypic data; (b) explore the metadata of primary investigations; (c) verify that primary data have overlapping genotypes; (d) find weather data that align temporally and spatially with the primary investigations; (e) reuse traits per genotype; and (f) aggregated traits with weather data.

**Fig. 1:**
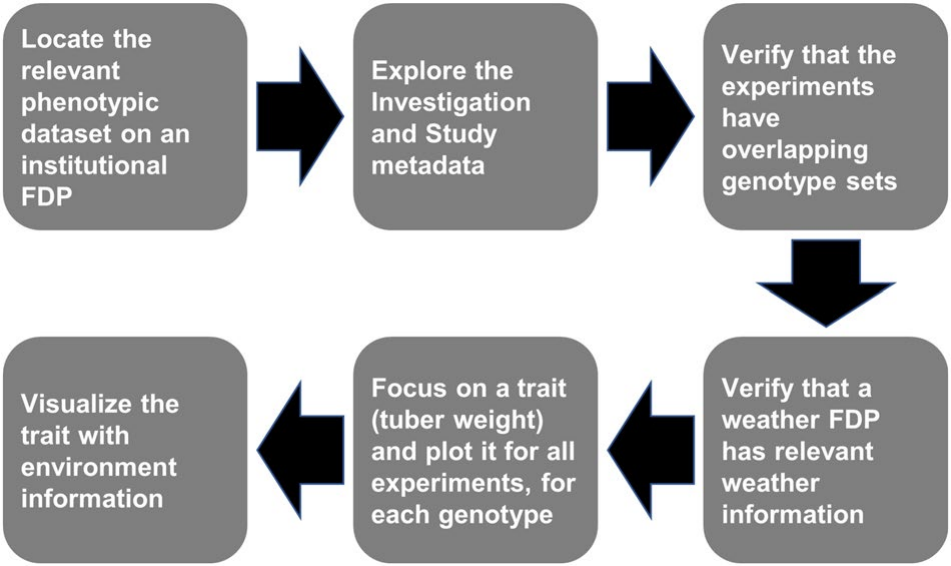
A diagram showing the steps that a researcher in this situation would follow to locate, acquire, examine, and reuse data in a FAIR way.

This work relies on the datasets that Hurtado-Lopez used in her doctoral thesis^11^, and more specifically on 5 phenotypic experiments and the respective photoperiod and temperature conditions at those locations. The Methods describe the process of making these datasets FAIR. We assume the role of a researcher who wants to conduct a multi-environment study with Hurtado-Lopez’s data. We navigate to the phenotypic datasets, check that necessary conditions are fulfilled, locate environmental (photoperiod and temperature) data, and combine them in an exploratory analysis. Though the underlying infrastructure is not fully developed for this proof of concept, the steps of discovery, integration and reuse rely on the FAIR principles (Fig. 1).

### Data

No phenotypic data was generated for the present work. The phenotyping experiments that were considered for this work were conducted in: the Netherlands (1999)^9^, Venezuela (2003), Finland (2004 and 2005)^10^, and Ethiopia (2010)^7^. All experimental data was retrieved from Hurtado-Lopez’s doctoral thesis supervisor after her departure from the university. Most data was relayed to Hurtado-Lopez by its original collectors and was subsequently organized by her, with the exception of the experiment in Ethiopia, which she co-supervised. It is unknown which, if any, steps were taken to disambiguate and harmonize the contents of these files. Hurtado-Lopez used weather data to conduct multi-environment and multi-trait analyses in her work^7^. Temperature and weather data was available through her files. In some cases, the resolution of the photoperiod datasets was too coarse, which we amended using an online source^12^. The data was available predominantly in the form of spreadsheets. The measurements for each experiment were recorded by different scientists, which is evident in the internal structure of the files. An overview of the datasets can be found in Table 1.

**Table 1:** Summary of the two datasets with their constituents and other attributes.

Dataset	Content description	Experiment ID	Types of source files
**Phenotypic dataset**	Data from the 1999 field trial in the Netherlands	1999NL	Excel files with experimental measurements
	Data from the 2003 field trial in Venezuela	2003VE	Excel files with experimental measurements
	Data from the 2004 field trial in Finland	2004Fin	Excel files with experimental measurements, genotype name translation files
	Data from the 2005 field trial in Finland	2005Fin	Excel files with experimental measurements, genotype name translation files
	Data from the 2005 field trial in Ethiopia	2010ET	Excel files with experimental measurements
**Weather dataset**	Photoperiod data (per day) for each location, covering at minimum the time period in question	N/A	Website (www.timeanddate.com)
	Temperature data (daily average) for each location, covering at minimum the time period in question	N/A	Excel files with measurements

### Process

We retrieved the field trial datasets, selected traits and placed the relevant data in tabular text files. We retrieved the metadata (from publications and local text documents, slidesets, and Hurtado-Lopez’s doctoral thesis), formatted it according to MIAPPE 1.1 and transformed it to RDF using PPEO^5^. Weather data was transformed using the AEMET weather ontology^13^.

For data owners to expose their (meta)data, and users to find and reuse the datasets, the FAIR Data Point (FDP) specification has been proposed^14^. FDPs present a hierarchy of levels (FDP, catalog, dataset, distribution) to organize and present their contents. Humans and machine agents can navigate FDPs, progressively harvesting (meta)data. We have constructed an FDP for this case^15^.

An observation was made during the configuration of the FDP Dataset metadata. The FDP specification recommends the use of certain attributes which mainly concern the resource itself. There is no support for domain-specific dataset annotations, which are necessary in our use case, since we need to be aware of the specifics of a dataset to reuse it, without having to look inside it (which is inefficient). Note that this limitation has been addressed in recent FDP implementation by adopting DCAT2, which facilitates interoperability between data catalogs on the Web^16^.

**Fig. 2:**
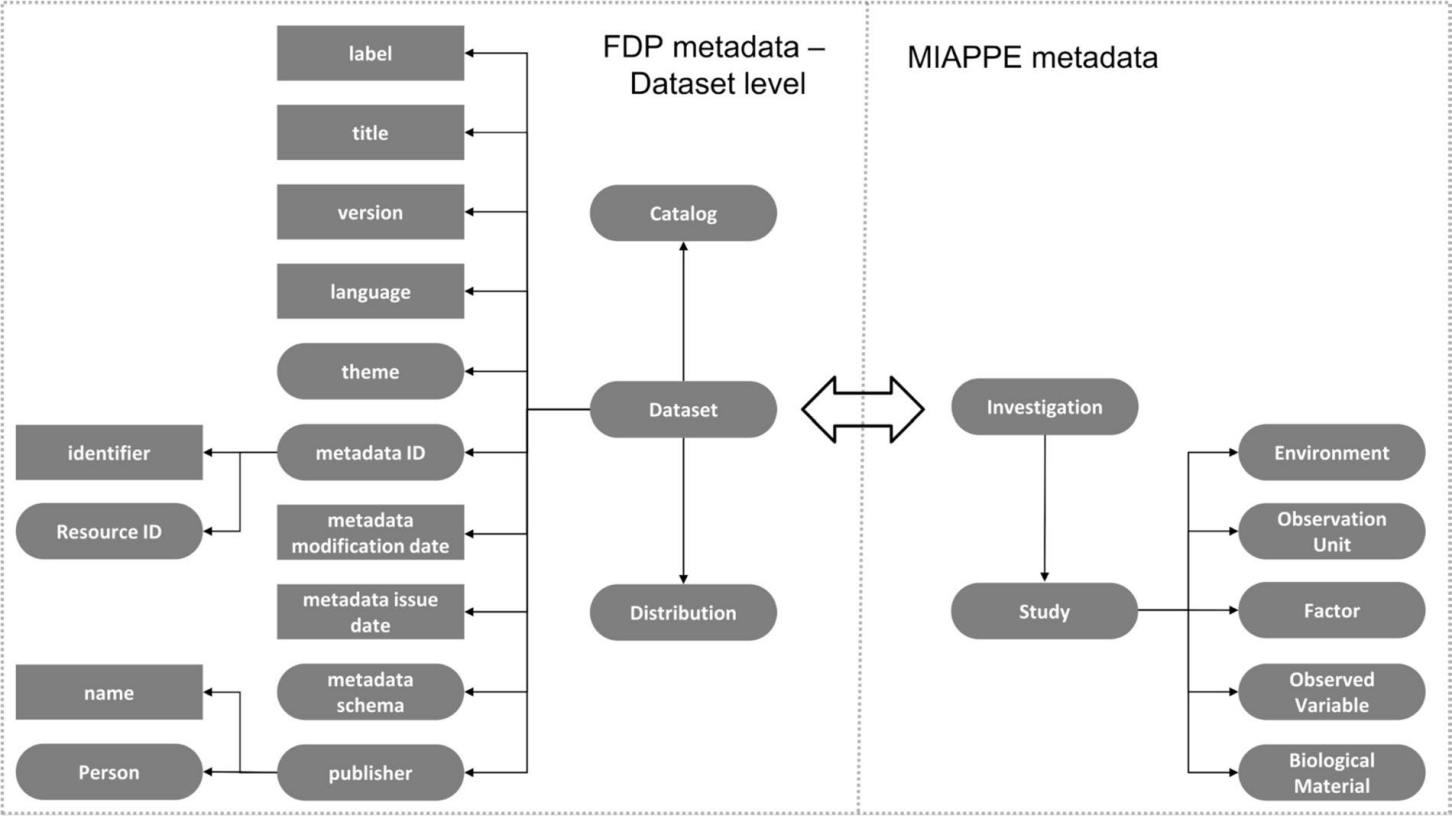
An illustration of the metadata specification Dataset level of the FDP (left), and our extension of it with MIAPPE metadata (right - incomplete). The FDP only includes metadata about the resource, which is not sufficient for describing the content of the dataset. Without concrete metadata about the dataset contents, the FDP cannot support meaningful, content-oriented indexing and searchability, so we have supplemented it with content-oriented MIAPPE metadata about the experimental data contained in the dataset. The dataset holds a MIAPPE Investigation, which is the connection point. Note only the connection that is made in the picture is between the Investigation and the Dataset. Other alignments between the two sides (e.g. Distribution and Study) have no meaning.

To support meaningful indexing and searchability on FAIR data portals, the FDP should provide metadata summarizing dataset contents. Our solution is extending the metadata descriptors that the FDP provides. We embedded the MIAPPE metadata into the Dataset level. The principle of the function of the FDP remains the same, but with this additional layer of metadata we can decide whether we are interested in a dataset without the additional step of accessing it. Figure 2 shows a schematic of this issue and our solution. The Methods section describes the FDP exploration in detail.

With the FDP in place, we can navigate to its topmost level and start exploring the data in a FAIR way. The role we assume is of a researcher who knows of the existence of the CxE datasets (though not their details), and wants to run a multi-environment, multi-trait analysis like Hurtado-Lopez. We did not conduct a biological analysis, as such methodologies are outside the scope of this work. Instead, we showcase what FAIR data discovery, acquisition, and integration could look like with six milestones (Fig. 1), each reflecting a step that a researcher could take in exploring and reusing the data.

For this scenario, the FDP lists a version (“distribution”) of the phenotypic dataset (including data and metadata) that is available as a triple store, and queriable with the SPARQL language.

This process is described in the Jupyter notebook, including all relevant files^15^. The section pointers below refer to the main notebook in that supplementary material (Explore_data.ipynb).

#### Finding relevant phenotypic datasets

**Fig. 3:**
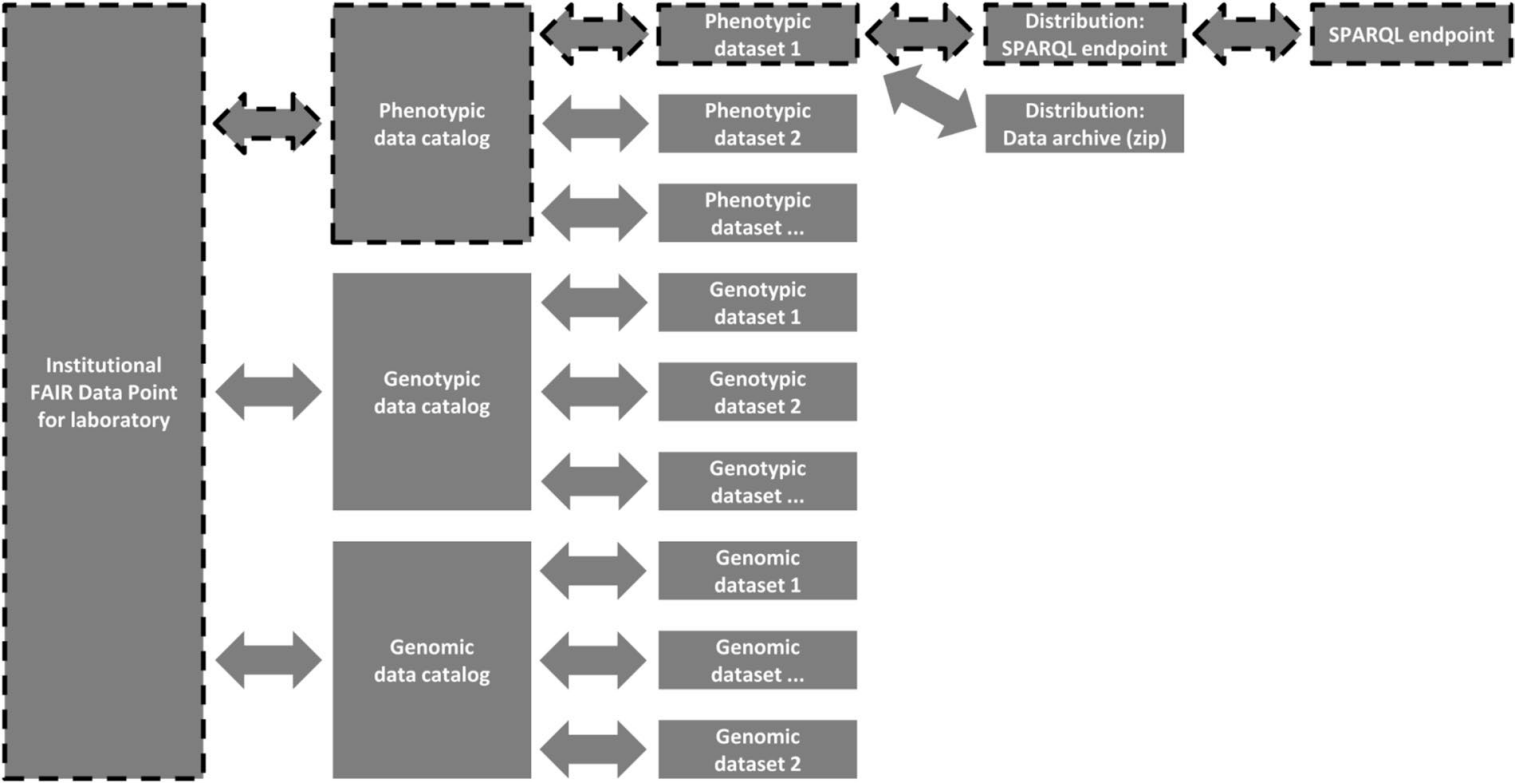
The structure of our FDP. One can start at the left and move toward the right, along the path indicated by the arrows, following the shapes with the dashed outlines. It is also possible to move backwards, as each level links to both its parent and its child.

To find relevant phenotypic datasets, a user navigates to the FDP of an institute. Its top level exposes metadata, among others, about the host institute and its data catalogs (Fig. 3). There are catalogs for three types of data here: phenotypic, genotypic, genomic. Because we are looking for the CxE phenotypic datasets, we navigate to the Phenotypic catalog. We examine each dataset, and we see that, out of the three present, one is relevant. To explore the dataset by querying it, we select the SPARQL endpoint distribution.

#### Exploring the investigation and Study metadata

**Fig. 4:**
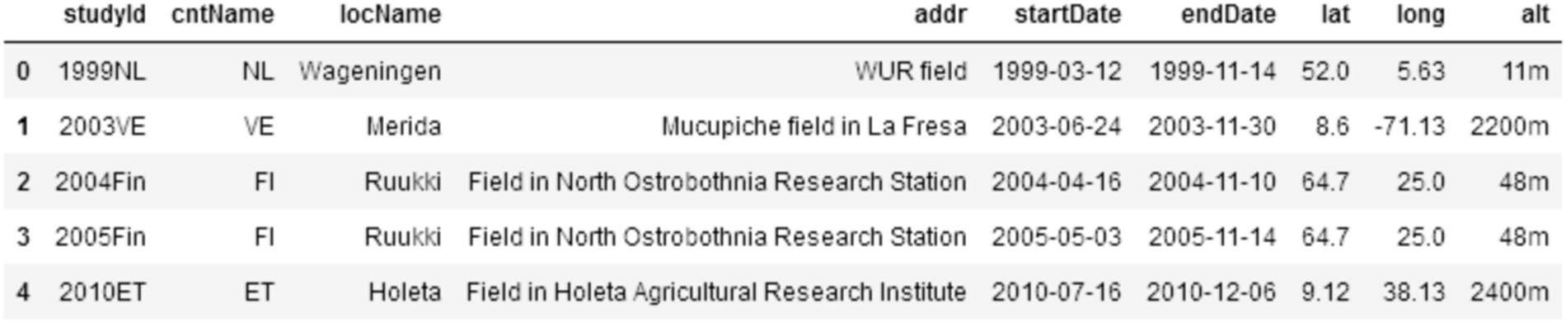
Fetching specific study values for each study: the study ID, the country abbreviation, the location name and address, the start and end dates of the study, its GPS coordinates and altitude (Notebook section 1.3).

We can write queries to find investigations and their properties. For a given investigation, we can then explore the studies it contains. A summary of the studies in this case is presented in Fig. 4.

#### Verifying that the experiments have overlapping genotypes

A prerequisite in this scenario (multi-environment analysis) is a genotype overlap in the studies. We verify this with a query. In total, there are 101 common genotypes across all experiments.

#### Finding weather data for the location and time period of interest

We previously discovered the address of a SPARQL endpoint holding the phenotypic data. We assume that we have similarly discovered the weather endpoint. We can query that and our phenotyping data endpoint in a federation, to ensure that the weather data we need is indeed there.

The weather data is structured around weather stations, each at given coordinates, producing measurements for weather variables. We have assigned a single weather station to each experimental location. In real life scenarios, the weather station coordinates would probably not overlap with those of the experimental fields.

**Fig. 5:**
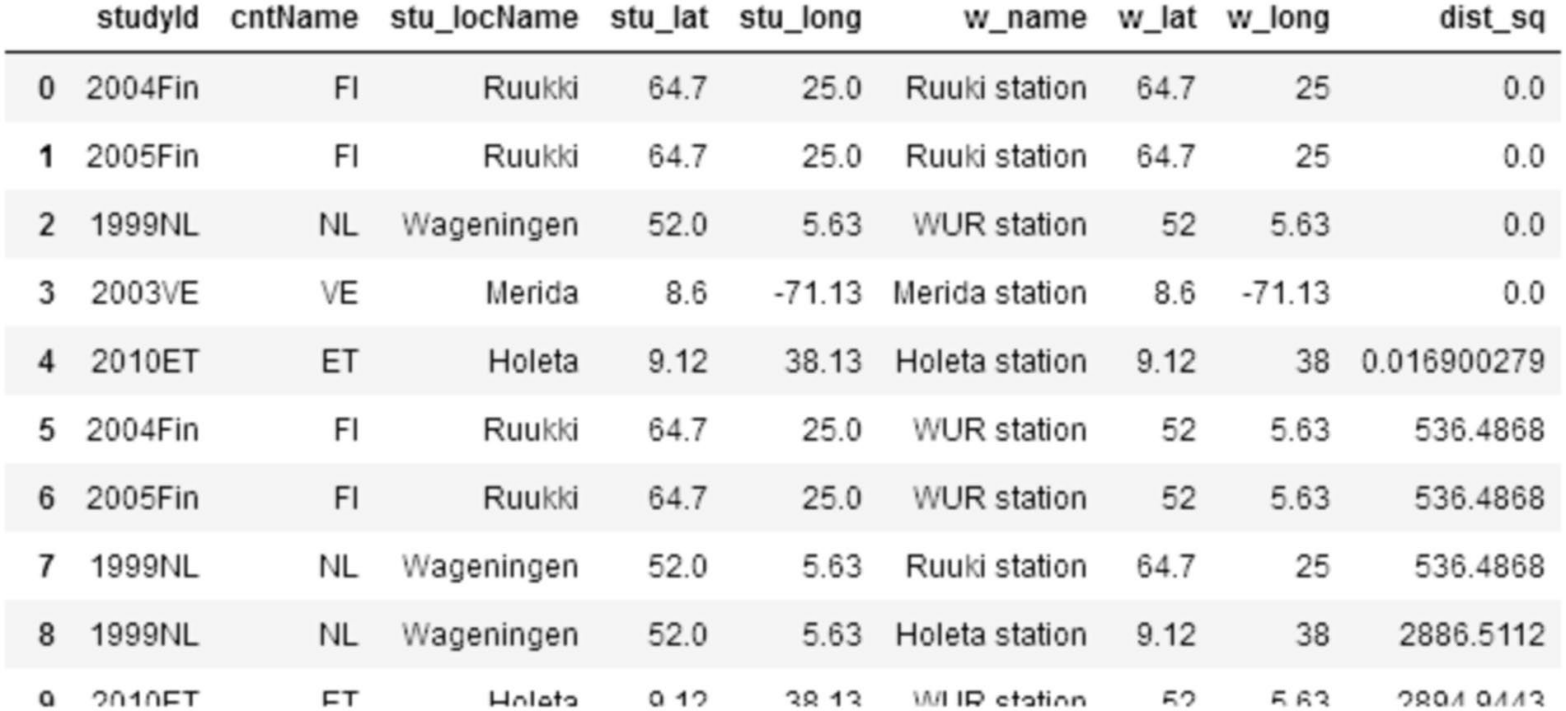
A query calculating the differences (squared) between the coordinates of each experiment and each weather station. Sorting the differences in ascending order confirms that there is a weather station that is suitable for each experiment (Notebook section 3.3).

The MIAPPE metadata gives us the location coordinates for the studies, from which we can calculate the distance to each weather station. Sorting in ascending order, we see the lowest values indicating the matches (Fig. 5).

#### Focusing on a trait and plotting it for all experiments, for each genotype

We will focus on the correlation between temperature and photoperiod, as Hurtado-Lopez’s doctoral thesis does (among other things), to demonstrate data integration.

**Fig. 6:**
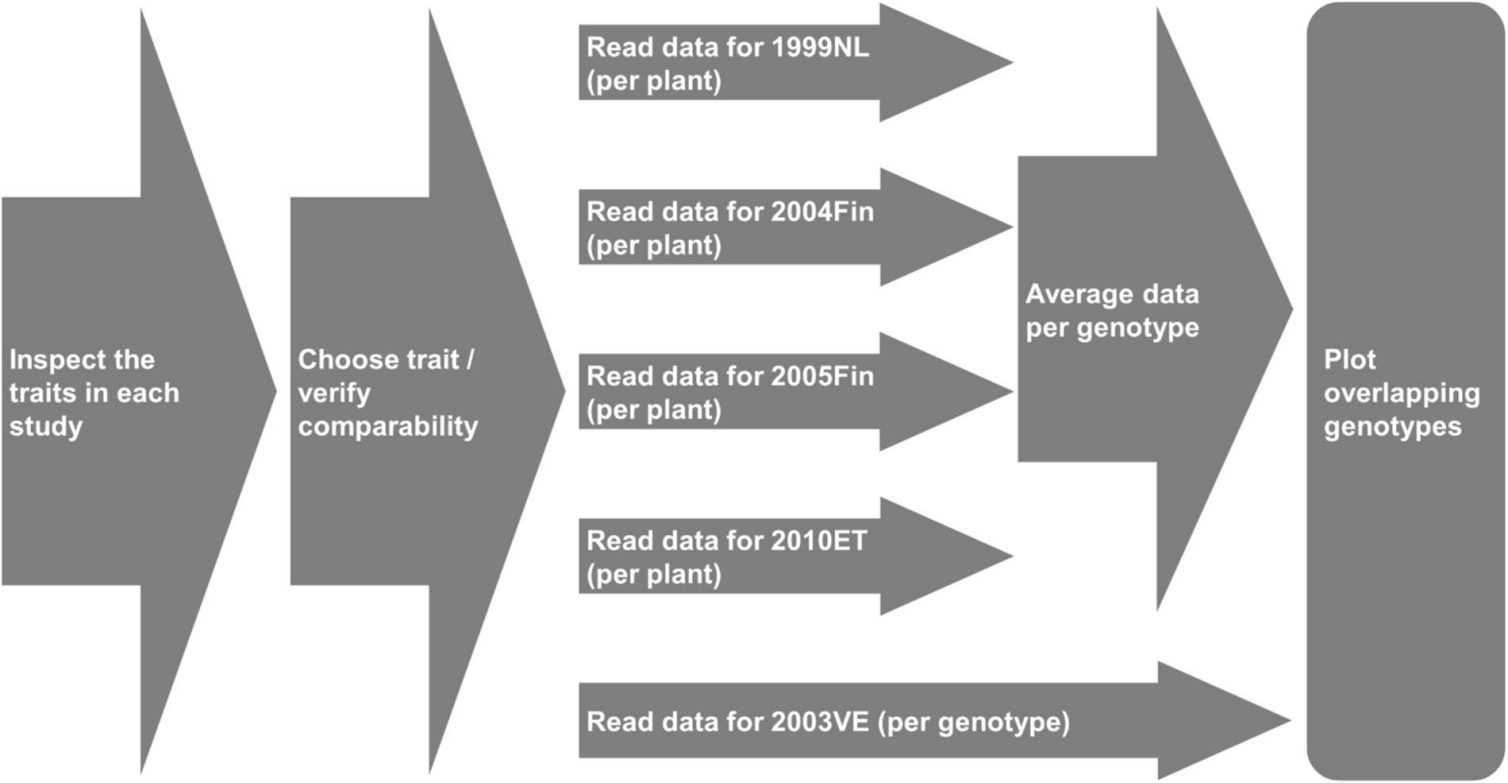
Necessary steps to be taken if a trait is to be compared/summarized in a plot across experiments.

A few traits are available for each study and they can be examined more closely through the observed variables. We will focus on the tuber weight per genotype for each experiment. The process for plotting a trait across all experiments is illustrated in Fig. 6.

**Fig. 7:**
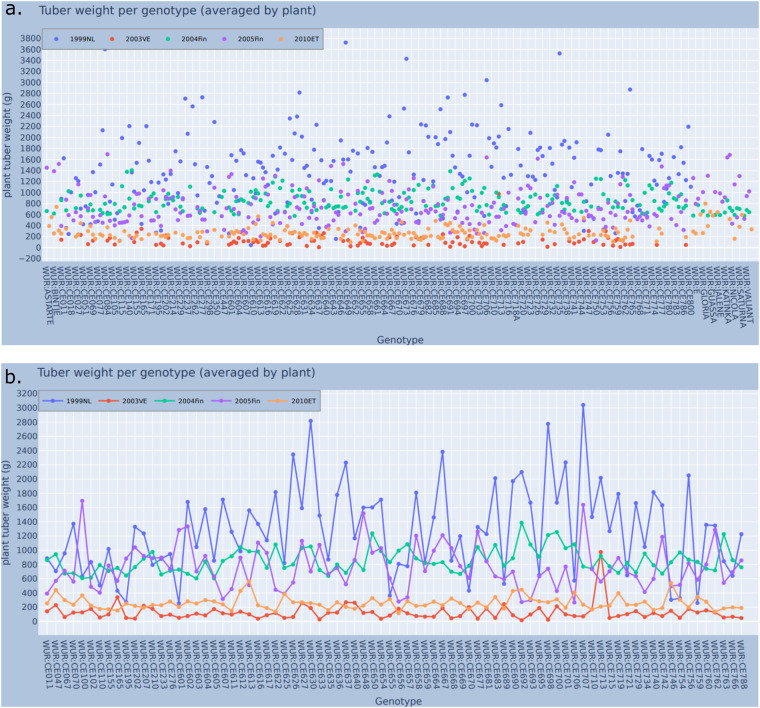
(**a**) A chart showing the total tuber weight produced, on average, by each genotype in each experiment, for a total of 292 genotypes (Notebook section 6). Not all genotypes have been used in all experiments. The x-axis on this chart lists the genotype name and the y-axis the average tuber weight per genotype (averaged by plant). The different dot colors show which experiment the data point corresponds to. (**b**) A chart showing the total tuber weight produced, on average, by each genotype in each experiment (Notebook section 5). Unlike the previous figure, this one includes the genotypes that not only were studied in all 5 experiments (101), but also had a value for our trait of interest (80). The x-axis on this chart lists the genotype name (though not all are labeled on the x-axis), and the y-axis the average tuber weight per plant per genotype. The different dot colors show which experiment the data point corresponds to.

The queries for assembling the data from the SPARQL endpoint are given in the Jupyter notebook (Section 4). In the responses, we see that there are sharp differences in the performance of the genotypes in each experiment, and great variability even for the same genotype. Figure 7a includes all genotypes (292), whereas Fig. 7b only the genotypes that were present in all five studies (101).

#### Making plots combining this trait with the weather information

In Hurtado-Lopez’s doctoral thesis, a measure combining the effects of temperature and photoperiod is used (as cumulative photo-beta thermal time - PBTT)^7^. With this data, we have calculated the PBTT values for each experiment as follows: 1999NL: 56.18; 2003VE: 23.11; 2004Fin: 31.31; 2005Fin: 39.3, 2010ET: 24.13.

We finally created a figure for tuber weight and PBTT. In it, we have combined three different data types: a) phenotypic (tuber weight values); b) experimental metadata (to get the genotypes associated to the plant IDs); and c) environmental (photoperiod).

**Fig. 8:**
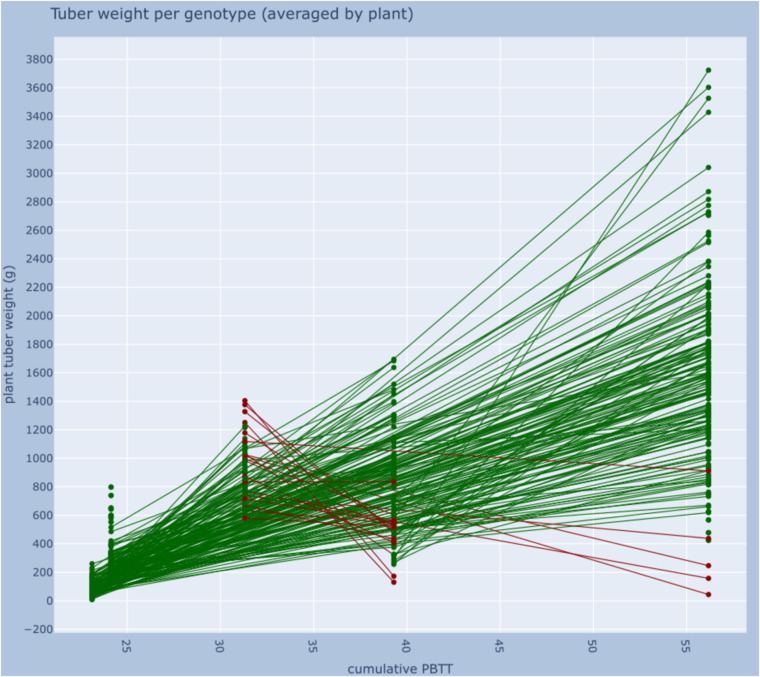
This figure shows the best and the worst performance for each genotype, depending on the environment (Notebook section 7.3). Each environment/study corresponds to a specific value of cumulative PBTT on the x-axis. The y-axis shows the average tuber weight per plant per genotype. A line that is rising (green) indicates that the genotype performed worst in an environment where the days were, on average short and the temperatures low, and better where days were long and temperatures high, whereas a falling line (red) indicates the opposite.

In Fig. 8, we draw a line for each genotype based on two data points: one for the lowest average tuber weight yielded by the plants of that genotype in an experiment, and one for the highest. Some lines rise and others fall; the difference between the minimum and maximum values can be seen as an indicator of the performance stability for that genotype.

For these results, we have used MIAPPE to ensure that key phenotypic data aspects are documented, standardized and discoverable on an FDP. Using an FDP makes it easier to integrate datasets not only in the same domain, but also across domains (e.g. phenotypic with environmental data). Although no new biological insight is given in these exploratory visualizations, this type of summary can be useful to indicate where particular attention is warranted by a domain expert.

## Discussion

The FAIR data principles encompass guidelines for data management to increase data reusability, which in turn is a necessary condition for reproducible science. In this work, we evaluate the domain-specific requirements for FAIR and the process to fulfill them in plant phenotyping. We demonstrate a pipeline with data already enriched with computer-readable documentation, and present a transparent pipeline that combines heterogeneous data across institutional and domain silos. The pipeline is composed of different stages for data discovery (Findability), acquisition (Accessibility /Interoperability), analysis and integration (Reusability) for exploratory purposes.

Most challenges revolved around interpreting (meta)data. The parties involved in data generation are frequently the only ones aware of details of experimental setups, as they are deemed obvious or not important enough to document/document in an organized manner. In this use case, the metadata was predominantly present as free text (in Hurtado-Lopez’s doctoral thesis and referenced papers). The scientists who originally collected data knew that, in order to publish their work and make it reproducible, the experiments had to be well-documented, and so made great efforts to provide all necessary information. In some cases, this work indicates that, in spite of those efforts, the results fell short of the ambition.

Ultimately, to resolve ambiguities and questions that arose and proceed with this work, it was necessary to consult with the coordinators of the original experiments on some details.

Even when the data files and the metadata were comprehensive and unambiguous, cleaning the original data files and reshaping them to a common format was time consuming, as was locating all relevant details in an ocean of free text. The process of (meta)data collection and harmonization was undertaken by a doctoral student with an engineering background who had spent three years interacting in the plant phenotyping community, helping improve data sharing methods in the domain. It should therefore be noted that the time investment may have been markedly different for a plant biologist or a scientist with a different background. In this work, it took on average two weeks per experiment to read the relevant literature, examine the pre-organized data files and reliably draw conclusions about the attributes that were collected. Furthermore, only a small fraction of the original data was harmonized due to time constraints. This work would have benefited appreciably from structured documentation, the likes of which have been unlikely in plant phenotyping until the establishment of MIAPPE: it would have effectively reduced this time investment to mere hours, as only a brief examination of the attributes and data transformation would have been required. The investment would have been lower for the scientists who originally collected and analyzed the data, as they are inherently familiar with their work.

We also discovered an issue with respect to findability. The FDP specification includes a “theme” that can be listed for a dataset, which can be as general as “science” or “biology”. For efficient reuse, further domain-specific, standardized details need to be present. There should be an overlap with the Minimum Information standards for different domains. Therefore, depending on the purpose, the definition of what should be “minimum” varies: whereas all MIAPPE details are relevant when it comes to describing a phenotyping experiment, not all of them are necessary to serve essential findability (e.g. observation unit details). The scenario illustrated here, with a human manually navigating an FDP, will not translate ideally to reality; instead, indexing FDPs should provide search functions over larger dataset collections, relying on minimum information that machines need to process and interpret.

From this work, it is obvious that there is a high knowledge barrier to entry when it comes to the technologies chosen here to implement FAIR. With graphical user interfaces, the steps of data discovery and acquisition could be facilitated greatly, so improving the user experience is one of the first points to be considered for improvement.

Our pipeline embraces all elements of FAIR. Findability is achieved with unique identifiers for every (meta)data element, connections between them and rich descriptors. Although the metadata is currently not findable through any community repository, the FDP structure combined with the (partial) metadata provides a clear path to doing so by indexing metadata for catalogs and datasets. For accessibility, we use a combination of established protocols (HTTP), data models (RDF) and formats (TTL), and ensure that metadata are available independently of the data. Reusability is achieved through the explicit provision of a license, MIAPPE, and data that follows emerging data formatting recommendations. Finally, MIAPPE and the use of a community-promoted implementation for it are responsible for (meta)data interoperability.

Despite the generality of the proof of concept, a key limitation of this work is that we conducted the proof of concept only once. This prohibits us to draw conclusions for a number of more generic issues related to FAIR. Such issues include, for example, whether it is worth making retrospectively data FAIR, or it should be reserved only for newly-collected data. Another important issue is to what extend a FAIRification process can be automated. To this we only offer our opinion, based on our experience.

It is imperative that the science community start the process of FAIR data sharing, even if it means making compromises on the technical side. A balance needs to be achieved between datasets that are FAIR-ready (i.e. equipped with good documentation and some identifiers) even though the technical provisions (FDPs, registries, curating, versioning and querying specifications) may not be fully in place. A first step is cultivating awareness in scientific communities that standards are beneficial and worth investment. This also requires a clear prioritization of historical datasets to be FAIRified in support of long-term experiments or for answering new questions^17,18^. Producing sufficient standards and using them to annotate one’s own datasets is time-consuming and other benefits (e.g. enhanced visibility and citability of one’s own datasets) have to be made clear. Furthermore, automating the FAIRification process may be possible when legacy data follow common formats, and in those cases it is worth to be considered. Last, but not least, data standards need to be promoted more strongly through the scientific publishing process, i.e. by journals, to create a basis for future incremental improvements.

## Methods

### Standards and data formats

MIAPPE has been in development by the plant phenotyping community and recently received enhancements to its specifications, formats and scope^5,19,20^. It is the standard for metadata provision in this community, and answers the explicit need for a domain-relevant community standard required by the FAIR data principles.

The MIAPPE implementations that are currently mature enough are the ISA-Tab format^21^, the Breeding API^6^ and the Plant Phenotype Experiment Ontology (PPEO)^22^. A spreadsheet implementation exists, though it is intended to play a secondary role to the others and serve as an easy way to introduce the standard to newcomers. For this work, a combination approach was deemed best: the spreadsheet has been used to assemble the metadata and as a means of intuitive inspection, and the PPEO to support machine readability.

As far as weather data is concerned, there is no clear preference for a specific ontology in the global community. The Linked Open Vocabularies portal^23^, which aggregates ontologies from the web so that they may be reused, presents three results to a “weather” query: the Home Weather ontology, the Smart Home Weather ontology, and the Air Traffic Data ontology. As they pertain to either smart home or air traffic contexts, they were not deemed appropriate. Further search indicated the BIMERR Weather Ontology^24^ and the SEAS Weather Ontology^25^ as possibilities, but no examples of actual use were found to support its adoption for this work. Contrary to that, the AEMET weather ontology^13^, of the Spanish Meteorological Office, is currently used to expose its data as linked data^13^, and for this reason it was chosen.

While MIAPPE does not specify a data format, it states that “The data files are formatted according to the common practices of the domain and contain references to that Variable ID, the measured values and times plus any information which researchers might deem useful”. We made the choice to use common tabular files with columns for the observation unit ID and measurement date, followed by the variable ID for the traits in question. In some cases, especially when it comes to time series, this results in sparse files. For the weather data, the same data format was followed.

### Technologies

#### FAIR data point (FDP)

To approach this work in a way that is consistent with the FAIR principles, it was necessary to produce a FAIR data point (FDP). The metadata was assembled according to the specifications developed by the Dutch Techcentre for Life Sciences^14^. It includes a tree structure with the metadata for the FDP itself at the root, followed by a Catalog (a collection of datasets), the dataset itself, and distributions of it (in different formats). Each level, on top of the metadata about itself, includes links to the ones below and above it, enabling navigation. The metadata is given in RDF/turtle format (ttl), and exposed by a python server script.

Part of the given details of a dataset, whether inside or outside an FDP, concern its contents or its description, so that interested parties may be aware of its topic prior to accessing it. In the general FDP specifications, a general way to accomplish this is indicated.

#### Resource description framework (RDF)

We chose to provide all data as linked RDF data. RDF is a W3C standard for data interchange, and one of the pillars powering the semantic web^26^. It is used to describe any kind of resource by making statements about it, each composed of three parts: a subject, a predicate and an object. Subjects are referred to by Unique Resource Identifiers (URIs) and statements can be made about them, connecting them to plain literals or other subjects with their own URIs. Semantic schemas specifying, for a given domain, possible ways to lay out and request information about subjects can be provided as ontologies. In turn, other parties can make statements about those subjects, eventually assembling a web of linked data.

RDF data can be queried using the SPARQL query language.

### FAIRification process

The process started with the FAIRification of the phenotyping datasets. Note that, for this work, no genotypic information is included. For this proof of concept only a few traits were chosen, with the goal to showcase FAIR data discovery, acquisition and integration.

The materials used are available in Github^15^.

#### Assembling the MIAPPE metadata

MIAPPE requires information for a number of categories. For each, an effort was made to locate the relevant information in P. Hurtado-Lopez’s thesis and related publications referenced therein. The information was compiled on MIAPPE’s spreadsheet format, using a different tab for each section.

- **Investigation:** We chose to represent the Investigation as a collection of the 5 experiments contained in P. Hurtado-Lopez’s thesis. The details reflect this content, and were chosen freely.
- **Study:** Each of the 5 phenotyping experiments comprises a study. General details, such as the location, start and end date and experimental design information, are listed here.
- **Person:** The coordinators of this work and P. Hurtado-Lopez are listed.
- **Data file:** 5 data files were composed, each holding the data for one study. They are listed here.
- **Biological material:** For this section, we create one biological material for each CxE genotype (including the parents), and other cultivars. In particular, the CxE cross is listed as the material source for each of the genotypes produced from it. It is important to maintain traceability of each genotype through the experiments, as we want to compare their performance in different environments.
- **Environment:** Details supplied in the thesis about the conditions (e.g. average temperature, type of soil) are present here, though this list is far from comprehensive.
- **Experimental factor:** There were no factors.
- **Events:** Planting dates, where known, are listed here, as well as applications of fungicides and known water treatments. Note that a date is necessary for the creation of an event, which prohibits the inclusion of un-timestamped occurrences. For example, in the case of fertilization events, sometimes fertilizer amounts are recorded, but not application dates. While this is relevant information, a time-stamped event cannot be created, and it rather needs to be included as an experimental factor.
- **Observation unit:** For most experiments, we have information about individual plants, and their organization into plots and blocks. The exception is the experiment in 2003 in Venezuela, where the data was first recorded (by the person who conducted this experiment) per plant, and then averaged for each genotype - which are the only data records communicated. In this case, the observation unit type “genotype” is used. There are some rare data points in other studies where the same unit type is used, as some genotypes were precluded from the records for not being a CxE clone.
- **Sample:** There were no samples to list.
- **Observed variable:** For the most part, the thesis gives a general description about the ways that plant traits were evaluated. In some cases, assumptions had to be made as there was ambiguity.

The resulting spreadsheet can be found on Github^15^.

The metadata documentation in MIAPPE is not always comprehensive. This can be observed in the sparsity of the Environment and Events section of the MIAPPE spreadsheet. For example, planting dates are mostly there, but the presence of only some actions around watering or fertilizer use highlights the information which was not recorded, and which could have been useful to know for future reuse. After all, the original data generator has no way of predicting which attributes may turn out significant for future re-users. Furthermore, whereas traits with complex evaluation procedures or scales are described in great detail, more “humble” ones don’t enjoy the same benefit. For example plant height measurements in wheat (*Triticum aestivum*) trials: It can be measured both “from the ground level to the tip of the spike”^27^ and “from the first node to the tip of the spike (excluding awns)”^28^ and therefore require transformations and other considerations if integration of such traits is required. When such details are unknown, reuse of data can be impossible or irresponsible.

Conflicts and ambiguities arise when the data is examined alongside the metadata. In some cases, the thesis/articles stated that, alongside a number of CE genotypes, commercial cultivars were planted and evaluated. The data itself may fail to include those (though in some cases may state that they have been removed), or fail to match the number of reported genotypes for that experiment.The files may include labels in different languages (presumably because of the authors’ native languages), use undocumented abbreviations (which renders the data invalid for reuse), or ones not specific enough (is this value a sum? an average? per plant or per plot? how many plants were averaged?). Some abbreviations had different meanings across files (e.g. “tottub” or presumably “total tubers”: in some cases it was the number per plant, in other cases the average for that genotype across plants) - which is crucial when one needs to be sure that the data points they wish to compare, from different experiments, are indeed compatible. Another complication arose from the use of symbols or categories outside the ones declared for certain traits (e.g. for a 0–7 flowering scale, undefined dots or asterisks).

#### Assembling the experimental data

**Fig. 9:**
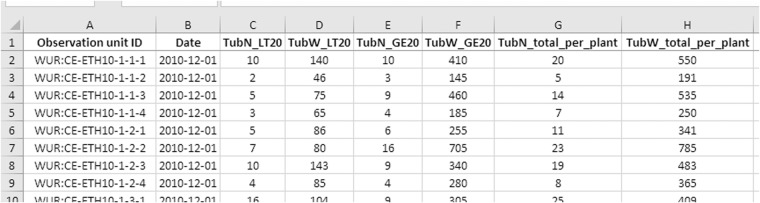
Part of the experimental data file for the 2010 Ethiopia experiment. The first column lists the observation unit ID, which can be cross-referenced with the metadata present in MIAPPE for more information, such as the observation level of that unit (in this case: plant), or its genotype. The date column is followed by columns labeled with the observation unit IDs, which again can be cross-referenced with MIAPPE metadata for a comprehensive explanation.

For each experiment, a tab-delimited text file was composed, holding all data for it. This includes data that was only measured once (e.g. total tuber weight for a plant), or as part of a time series. An example of such a file can be seen in Fig. 9.

#### Generating RDF

The MIAPPE spreadsheet holding the information about the experiments was processed using a Python script. This script used PPEO to produce RDF from the spreadsheet data, capturing all of its contents. The process can be inspected in a Jupyter notebook. Note that certain formatting assumptions were made (for example, for the location details cells of the spreadsheet) to make this possible. This process is fully reproducible, however this is not a general-purpose converter as it does not cover the parts of MIAPPE (sections, attributes) that were not used in this work.

PPEO was also used to generate RDF for the actual experimental data.

All generated RDF files are available in our data repository^29^.

#### Exposing RDF

The lightweight Jena Fuseki triple store was chosen to serve as a SPARQL endpoint. In it, two datasets were created: one for the phenotypic data, and one for the weather data. In practice, these can be treated as two separate SPARQL endpoints and they can be queried individually. In a real life scenario, it would be realistic to have a long list of SPARQL endpoints, but two are sufficient for the purposes of an integration showcase. With federation, these two endpoints can collaborate and respond to questions that address data on both - whereas each individual one cannot.

For the FDP, TTL files were composed with relevant metadata, and then exposed as plain text using a Python script (with the Flask library).

### Exploring the FDP

For this, the user has to navigate to the FDP of their institute, that holds a list of catalogs belonging to this FDP, and in this case there is one catalog for each domain. Since they are looking for a phenotypic dataset, the user can therefore navigate to the phenotypic data catalog.

**Fig. 10:**
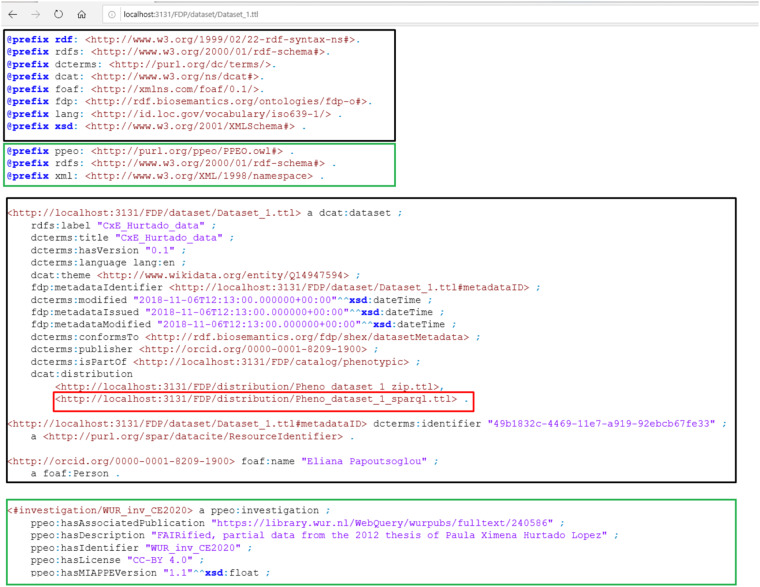
Metadata for dataset 1. The black frames indicate the FDP dataset metadata specification. Everything else (green frames) is from MIAPPE (incomplete) and has been added here to give an indication as to the specific contents of this dataset. Finally, following the link to the SPARQL distribution, we find the URL of a SPARQL endpoint hosting the dataset of interest. We can use this to start exploring it.

The catalog itself includes a number of datasets. No further information is given on this page about them, so they have to be manually checked. The one they are interested in turns out to be Dataset 1, the metadata for which is shown on Fig. 10. It should be noted that the FDP specification contains no recommendations about the description of datasets. Therefore, to provide essential metadata about the contents of this phenotypic dataset (biological materials, observed variables, etc.), we have supplemented it with MIAPPE metadata (a schematic view is given in Fig. 2. Because of this supplementation, if this FDP were to be indexed, information about the actual dataset contents could be harvested and enable content-related searches using the MIAPPE vocabulary. The metadata page of Dataset 1, shown in Fig. 10, is composed of two parts: (a) generic dataset-level metadata in the FDP specification for the dataset level; and (b) domain-specific dataset-level metadata, using MIAPPE.

## Data Availability

All associated data, original and processed, is available on Github^15^. The data is located under the paths “all_containers/ common_files/data-original” and “all_containers/common_files/data-generated”. These two folders (data-original and data-generated) are also available on Zenodo^29^.

## Code availability

All associated code is available on our Github repository and deposited on Zenodo^15^, including Jupyter notebooks to transform data, scripts to run the FDP and the triple store.
